# Prognosis prediction performs better in patients with non-cirrhosis hepatitis B virus-related acute-on-chronic liver failure than those with cirrhosis

**DOI:** 10.3389/fmicb.2022.1013439

**Published:** 2022-12-09

**Authors:** Xia Yu, Hai Li, Wenting Tan, Xianbo Wang, Xin Zheng, Yan Huang, Beiling Li, Zhongji Meng, Yanhang Gao, Zhiping Qian, Feng Liu, Xiaobo Lu, Jia Shang, Huadong Yan, Yubao Zheng, Weituo Zhang, Shan Yin, Wenyi Gu, Guohong Deng, Xiaomei Xiang, Yi Zhou, Yixin Hou, Qun Zhang, Shue Xiong, Jing Liu, Ruochan Chen, Liyuan Long, Jinjun Chen, Xiuhua Jiang, Sen Luo, Yuanyuan Chen, Chang Jiang, Jinming Zhao, Liujuan Ji, Xue Mei, Jing Li, Tao Li, Rongjiong Zheng, Xinyi Zhou, Haotang Ren, Jifang Sheng, Yu Shi

**Affiliations:** ^1^State Key Laboratory for Diagnosis and Treatment of Infectious Diseases, National Clinical Research Center for Infectious Diseases, Collaborative Innovation Center for Diagnosis and Treatment of Infectious Diseases, The First Affiliated Hospital, College of Medicine, Zhejiang University, Hangzhou, China; ^2^Department of Gastroenterology, Renji Hospital, School of Medicine, Shanghai Jiao Tong University, Shanghai, China; ^3^Shanghai Institute of Digestive Disease, Key Laboratory of Gastroenterology and Hepatology, Chinese Ministry of Health, Shanghai Jiao Tong University, Shanghai, China; ^4^Department of Infectious Diseases, Southwest Hospital, Third Military Medical University (Army Medical University), Chongqing, China; ^5^Center of Integrative Medicine, Beijing Ditan Hospital, Capital Medical University, Beijing, China; ^6^Department of Infectious Diseases, Institute of Infection and Immunology, Union Hospital, Tongji Medical College, Huazhong University of Science and Technology, Wuhan, China; ^7^Department of Infectious Diseases, Hunan Key Laboratory of Viral Hepatitis, Xiangya Hospital, Central South University, Changsha, China; ^8^Hepatology Unit, Department of Infectious Diseases, Nanfang Hospital, Southern Medical University, Guangzhou, China; ^9^Department of Infectious Diseases, Hubei Clinical Research Center for Precise Diagnosis and Treatment of Liver Cancer, Taihe Hospital, Hubei University of Medicine, Shiyan, China; ^10^Department of Hepatology, The First Hospital of Jilin University, Changchun, China; ^11^Department of Liver Intensive Care Unit, Shanghai Public Health Clinical Center, Fudan University, Shanghai, China; ^12^Tianjin Institute of Hepatology, Nankai University Second People’s Hospital, Tianjin, China; ^13^Department of Infectious Diseases and Hepatology, The Second Hospital of Shandong University, Jinan, China; ^14^Infectious Disease Center, The First Affiliated Hospital of Xinjiang Medical University, Ürümqi, China; ^15^Department of Infectious Diseases, Henan Provincial People’s Hospital, Zhengzhou, China; ^16^Department of Infectious Diseases, Shulan (Hangzhou) Hospital Affiliated to Zhejiang Shuren University, Shulan International Medical College, Hangzhou, China; ^17^Department of Infectious Diseases, The Third Affiliated Hospital of Sun Yat-sen University, Guangzhou, China; ^18^Clinical Research Center, Shanghai Jiao Tong University School of Medicine, Shanghai, China

**Keywords:** hepatitis B virus, acute-on-chronic liver failure, clinical prediction models, cirrhosis, performance

## Abstract

**Background:**

The accurate prediction of the outcome of hepatitis B virus-related acute-on-chronic liver failure (HBV-ACLF) is impeded by population heterogeneity. The study aimed to assess the impact of underlying cirrhosis on the performance of clinical prediction models (CPMs).

**Methods:**

Using data from two multicenter, prospective cohorts of patients with HBV-ACLF, the discrimination, calibration, and clinical benefit were assessed for CPMs predicting 28-day and 90-day outcomes in patients with cirrhosis and those without, respectively.

**Results:**

A total of 919 patients with HBV-ACLF were identified by Chinese Group on the Study of Severe Hepatitis B (COSSH) criteria, including 675 with cirrhosis and 244 without. COSSH-ACLF IIs, COSSH-ACLFs, Chronic Liver Failure-Consortium Acute-on-Chronic Liver Failure score (CLIF-C ACLFs), Tongji Prognostic Predictor Model score (TPPMs), Model for End-Stage Liver Disease score (MELDs), and MELD-Sodium score (MELD-Nas) were all strong predictors of short-term mortality in patients with HBV-ACLF. In contrast to a high model discriminative capacity in ACLF without cirrhosis, each prognostic model represents a marked decline of C-index, net reclassification index (NRI), and integrated discrimination improvement (IDI) in predicting either 28-day or 90-day prognosis of patients with cirrhosis. The hazard analysis identified largely overlapping risk factors of poor outcomes in both subgroups, while serum bilirubin was specifically associated with short-term mortality in patients with cirrhosis and blood urea nitrogen in patients without cirrhosis. A subgroup analysis in patients with cirrhosis showed a decline of discrimination of CPMS in those with ascites or infections compared to that in those without.

**Conclusion:**

Predicting the short-term outcome of HBV-ACLF by CPMs is optimal in patients without cirrhosis but limited in those with cirrhosis, at least partially due to the complicated ascites or infections.

## Introduction

Acute-on-chronic liver failure (ACLF) is a critical illness caused by the acute exacerbation of a chronic liver disease, which leads to organ failure(s) and high short-term mortality (Bernal et al., 2015; Hernaez et al., 2017). The etiologies of ACLF differ in countries and regions across the world. In the Asian Pacific region, especially in China, HBV-related ACLF remains to be the major subtype of ACLF and one of the main causes of death in individuals with chronic HBV infection (Shi et al., 2015b).

Due to the shortage of donor organs and high mortality on the waiting list, it is of paramount importance at hospital admission to differentiate patients who would die in a short-term period despite standard care from those who would recover. Many efforts have been put to utilize clinical prediction models (CPMs) in the prognostication of HBV-ACLF, including non-specific scoring models [the model for end-stage liver disease (MELD) (Malinchoc et al., 2000) and Child–Turcotte–Pugh (CTP) score (Pugh et al., 1973)] and those specifically developed for HBV-ACLF (Yan et al., 2015). Among them, the MELD score was the most widely assessed (Wong and Cai, 2005). A wide variation in MELD performance was reported among different studies which represents significant heterogeneity of study populations (Yu et al., 2021). It is speculated that the presence of liver cirrhosis would be an important variable affecting the predictive accuracy of one specific prognostic model, as indicated by the fact that non-cirrhotic ACLF displays distinct patterns of organ failures from those with cirrhosis (Arroyo et al., 2016). While current reported studies have focused on prognostic factors of various types and forms of ACLF (Yu et al., 2021), how the presence of cirrhosis affects the performance of a clinical predictive model for patients with HBV-ACLF remained unknown. To confirm the hypothesis, we aimed to assess the accuracy of selected scoring models [that were MELD (Malinchoc et al., 2000) and MELD-Sodium score (MELD-Nas) (Biggins et al., 2006) representative of current donor organ allocation systems, Chronic Liver Failure-Consortium Acute-on-Chronic Liver Failure Score (CLIF-C ACLFs) (Jalan et al., 2014b) representative of score systems for general ACLF population, Tongji Prognostic Predictor Model score (TPPMs) (Wang et al., 2014), Chinese Group on the Study of Severe Hepatitis B (COSSH-ACLF), (Wu et al., 2018) and COSSH-ACLF IIs (Li et al., 2021) representative of score systems specific for HBV-ACLF] in predicting the short-term mortality of HBV-ACLF with or without cirrhosis, respectively, using data from a large multicenter, prospective cohort [the Chinese Acute-on-Chronic Liver Failure (CATCH-LIFE)] study (Gu et al., 2018; Qiao et al., 2021).

## Materials and methods

### Study design and population

We retrospectively used data from two prospective multicenter cohorts of the CATCH-LIFE study (NCT02457637 and NCT03641872) from January 2015 to December 2016 and September 2018 to January 2019. The CATCH-LIFE study was designed to investigate the natural history of patients with chronic liver disease and acute exacerbation. The multicenter study is held by the Chinese Chronic Liver Failure (CLIF) Consortium, which is composed of 15 tertiary hospitals in China [Renji Hospital, School of Medicine, Shanghai Jiao Tong University, Shanghai; Southwest Hospital, Third Military Medical University, Chongqing; Wuhan Union Hospital, Tongji Medical College, Huazhong University of Science and Technology, Hubei; Nanfang Hospital, Southern Medical University, Guangzhou; Beijing Ditan Hospital, Capital Medical University, Beijing; Xiangya Hospital, Central South University, Hunan; First Hospital of Jilin University (JU), Jilin; Taihe Hospital, Hubei University of Medicine, Hubei; Shanghai Public Health Clinical Centre (SPHCC), Fudan University, Shanghai; Second Hospital of Shandong University (SDU), Shandong; First Affiliated Hospital of Xinjiang Medical University (XMU), Xinjiang; Henan Provincial People’s Hospital, Henan; Tianjin, Affiliated Hospital of Logistics University of People’s Armed Police Force, Tianjin; Fuzhou General Hospital of Nanjing Military Command, Fujian; and The First Affiliated Hospital of Zhejiang University in Zhejiang province, Zhejiang].

The patient inclusion criteria of the CATCH-LIFE study were: (1) inpatients (length of stay >1 day), including patients in the emergency observation ward; (2) patients with chronic liver disease including patients with non-alcoholic fatty liver disease, patients with chronic hepatitis without cirrhosis, and patients with compensated/decompensated cirrhosis; and (3) patients with acute liver injury (ALI) (ALT or AST > 3 × Upper normal limit or total bilirubin > 2 × Upper normal limit (within 1 week before enrollment) or acute decompensation (AD) event(s) (ascites, hepatic encephalopathy, bacterial infection, or gastrointestinal bleeding within 1 month before enrollment). The exclusion criteria were: (1) age ≤15 or ≥80; (2) pregnant women; (3) hepatocellular carcinoma or other liver malignancies were detected before or during the first admission; (4) malignancies in other organs; and (5) severe chronic extrahepatic disease. With these criteria, we further identified patients with HBV-ACLF in the CATCH-LIFE cohort by applying the Chinese Group on the Study of Severe Hepatitis B (COSSH) diagnostic criteria (Wu et al., 2018). In addition, to exclude the heterogeneity of the included patients with HBV-ACLF by different diagnostic criteria, we further adapted the APASL consensus diagnostic criteria (Sarin et al., 2009, 2014, 2019) to include patients with HBV-ACLF to verify the conclusion. Cirrhosis was diagnosed by endoscopic signs of portal hypertension or radiological evidence of liver nodularity in patients with chronic liver diseases as previously described (Tsochatzis et al., 2014). Chronic HBV infection is defined as a prolonged serum HBsAg positivity for 6 months.

After enrollment, demographic, laboratory, radiological, and other clinical information were collected for each patient. After discharge, patients were followed up through outpatient records, telephone, or WeChat. Death or liver transplantation was recorded during follow-up. The primary endpoint of the study was death within 28 days and 90 days post-enrollment.

The study adheres to the Declaration of Helsinki and was approved by the Renji Hospital Ethics Committee of Shanghai Jiao Tong University School of Medicine, and written consent was obtained from all the study patients or their legal representatives.

### Scoring systems

We selected COSSH-ACLF IIs and COSSH-ACLFs representative of CPMs specific for HBV-ACLF, CLIF-C ACLFs for ACLF, MELD, and MELD-Nas for end-stage liver diseases. The COSSH-ACLF IIs calculation formula is as follows: 1.649 × ln (international normalized ratio) + 0.457 × hepatic encephalopathy score + 0.425 × ln (neutrophil) + 0.396 × ln (total bilirubin) + 0.576 × ln (serum urea) + 0.033 × age (Li et al., 2021). The COSSH-ACLFs calculation formula is as follows: 0.741 × INR + 0.523 × HBV − sequential organ failure assessment score (SOFAs) + 0.026 × age + 0.003 × TB (μmol/L) (Wu et al., 2018). The CLIF-C ACLFs calculation formula is as follows: 10 × {0.33 × CLIF − organ failure (OFs) score + 0.04 × age + 0.63 × ln [white blood cell (WBC)) − 2} (Jalan et al., 2014b). The TPPMs calculation formula is as follows: *P* = 1 / (1 + e^–logit(^*^P^*^)^), logit(*P*) = 0.003 × [TBil (μmol/L)] + 0.951 × INR + 2.258 × [constant for complications: 0 if without or with one complication; 1 with 2 or more complications] + 0.114 × [lg HBV DNA (copies/ml)] − 5.012 (Wang et al., 2014). The MELDs calculation formula is as follows: 3.78 × ln [TB (mg/dl)] + 11.2 × ln (INR) + 9.57 × ln [serum creatinine (mg/dl)] + 6.43 (Malinchoc et al., 2000). The MELD-Nas calculation formula is as follows: MELD + 1.59 (135–serum sodium) (Biggins et al., 2006).

### Statistical analysis

Continuous variables were expressed as mean ± standard deviation (SD) or median (range), and categorical variables were expressed as counts (percentage). Student’s *t*-test or the Mann–Whitney U-test was used for the comparison of continuous variables, and χ^2^-test was used for the comparison of categorical variables. The 28-day and 90-day survival of HBV-ACLF patients with or without cirrhosis were shown by the cumulative correlation function (CIF) following a Fine–Gray competing risk model, in which the liver transplantation (LT) was regarded as a competing event with death (Fine and Gray, 1999). The hazard ratio (HR) of each scoring model associated with death was estimated by a Cox proportional hazards regression model.

The performance of scoring models in overall patients and cirrhosis/non-cirrhosis subgroups was compared in aspects of discrimination and calibration. The discrimination of models was measured by the C-index, integrated discrimination improvement (IDI), and the net reclassification improvement (NRI) metric. A C-index of >0.80 indicates a good discriminative performance of a prognostic model. An NRI or IDI of >0 indicates the improvement of discrimination in the new model over the reference model. The calibration of models was assessed by the Hosmer–Lemeshow (H–L) test, a calibration plot, Nagelkerke’s *R*^2^, and the Brier score. For the H–L test, the smaller the χ^2^, the greater the correlation *p*-value and the better the goodness-of-fit. Suitable calibration is indicated by an H–L *p*-value of ≥0.05. A higher *R*^2^ and a lower Brier score indicate better calibration. In addition, the clinical benefit of scoring models was evaluated by decision curve analysis (DCA). All the statistical analyses were undertaken with R software (version 4.0.5; The R Foundation for Statistical Computing^1^), and differences were considered significant at a *p*-value of <0.05.

## Results

### Baseline characteristics of patients

**FIGURE 1:**
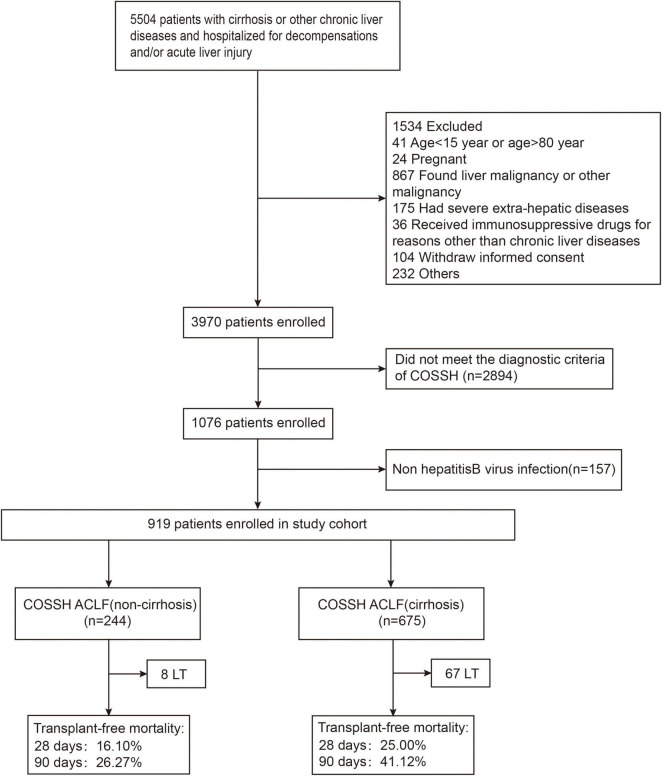
The flowchart of study selection. LT, liver transplantation; COSSH, Chinese Group on the Study of Severe Hepatitis B.

As shown in Figure 1 and Table 1, a total of 919 patients with HBV-ACLF were included after excluding patients who were not eligible, with 675 with cirrhosis and 244 without. HBV-ACLF patients with cirrhosis were elder than those without cirrhosis (48 ± 11 vs. 43 ± 12, *p* < 0.001). They were more likely to develop gastrointestinal bleeding [39 (5.8%) vs. 0 (0%), *p* < 0.001], ascites [456 (69.0%) vs. 87 (35.7%), *p* < 0.001], and infection [258 (38.2%) vs. 62 (25.4%), *p* < 0.001). Patients with cirrhosis were more likely to develop renal failure [58 (8.6%) vs. 7 (2.9%), *p* = 0.003] and coagulation failure [256 (37.9%) vs. 75 (30.7%), *p* = 0.045] than patients without cirrhosis. As to the laboratory tests, patients with cirrhosis had a lower level of albumin (g/L) [30.6 (6.8) vs. 32.6 (6.2), *p* < 0.001], alanine aminotransferase (ALT) (U/L) [173.7 (436.0) vs. 602.8 (973.0), *p* < 0.001], aspartate aminotransferase (AST) (U/L) [185.2 (315.0) vs. 367.0 (626.2), *p* < 0.001], glutamyl transferase (GGT) (U/L) [72.0 (74.2) vs. 87.2 (75.0), *p* = 0.004], sodium (mmol/L) [136.0 (7.0) vs. 137.0 (5.2), *p* < 0.001], hemoglobin [120.0 (29.0) vs. 133.0 (26.0), *p* < 0.001], and platelets (10^9^/L) [83.0 (61.0) vs. 120.5 (65.0), *p* < 0.001] than without cirrhosis. In contrast, patients with cirrhosis had a higher level of creatinine (μmol/L) [74.0 (38.8) vs. 68.0 (26.7), *p* < 0.001], urea nitrogen (mmol/L) [4.8 (3.9) vs. 3.7 (1.8), *p* < 0.001], higher neutrophil-to-lymphocyte ratio (NLR) [4.0 (4.3) vs. 3.3 (3.2), *p* = 0.030], and higher international normalized ratio (INR) [2.1 (1.0) vs. 2.0 (0.8), *p* = 0.025]. In addition, patients with cirrhosis had higher disease risk scores including COSSH-ACLF IIs [7.2 (1.2) vs. 6.7 (1.2), *p* < 0.001], COSSH-ACLFs [7.2 (1.8) vs. 6.3 (1.5), *p* < 0.001], CLIF-C ACLFs [40.5 (8.6) vs. 37.6 (10.3), *p* < 0.001], TPPMs [0.4 (0.6) vs. 0.2 (0.3), *p* < 0.001], MELDs [27.1 (7.0) vs. 25.4 (5.5), *p* < 0.001], and MELD-Nas [28.9 (10.6) vs. 26.3 (6.3), *p* < 0.001].

**TABLE 1:** Patient characteristics at enrollment.

Variables	Total HBV-ACLF (*n* = 919)	HBV-ACLF with cirrhosis (*n* = 675)	HBV-ACLF without cirrhosis (*n* = 244)	*P-*value
Age (years)	47 ± 11	48 ± 11	43 ± 12	<0.001
**Sex**				
Male	792 (86.2%)	590 (87.4%)	202 (82.8%)	
Female	127 (13.8%)	85 (12.6%)	42 (17.2%)	0.073
MAP (mmHg)	89 ± 11	89 ± 11	89 ± 10	0.965
**Etiology**				
HBV	689 (75.0%)	516 (76.4%)	173 (70.9%)	
HBV + alcohol	128 (13.9%)	96 (14.2%)	32 (13.1%)	
HBV + others	102 (11.1%)	63 (9.3%)	39 (16.0%)	0.018
**Precipitating event**				
Hepatic insults alone	210 (22.9%)	141 (20.9%)	69 (28.3%)	
Mixed with extrahepatic insults	122 (13.3%)	99 (14.7%)	23 (9.4%)	
Extrahepatic insults alone	187 (20.3%)	154 (22.8%)	33 (13.5%)	
Unknown	400 (43.5%)	281 (41.6%)	119 (48.8%)	0.001
**Complications**				
GIH	39 (4.2%)	39 (5.8%)	0 (0%)	<0.001
Ascites	553 (60.2%)	456 (69.0%)	87 (35.7%)	<0.001
HE	120 (13.1%)	91 (13.5%)	29 (11.9%)	0.526
Infection	320 (34.8%)	258 (38.2%)	62 (25.4%)	<0.001
SBP	24 (2.6%)	22 (3.3%)	2 (0.8%)	0.041
Pneumonia	222 (24.2%)	176 (26.1%)	46 (18.9%)	0.024
Other infection	86 (9.4%)	72 (10.7%)	14 (5.7%)	0.023
**Laboratory data**				
Alb (g/L)	31.0 (6.7)	30.6 (6.8)	32.6 (6.2)	<0.001
ALT (U/L)	241.0 (591.3)	173.7 (436.0)	602.8 (973.0)	<0.001
AST (U/L)	211.0 (384.7)	185.2 (315.0)	367.0 (626.2)	<0.001
AKP (U/L)	144.0 (72.9)	142.0 (73.0)	149.0 (74.0)	0.331
TB (μmol/L)	343.8 (235.5)	350.0 (216.2)	328.1 (210.8)	0.076
GGT (U/L)	77.0 (73.3)	72.0 (74.2)	87.2 (75.0)	0.004
Cr (μmol/L)	72.0 (37.0)	74.0 (38.8)	68.0 (26.7)	<0.001
BUN (mmol/L)	4.4 (3.1)	4.8 (3.9)	3.7 (1.8)	<0.001
K (mmol/L)	3.9 (0.8)	3.9 (0.8)	3.9 (0.7)	0.574
Na (mmol/L)	136.7 (6.2)	136.0 (7.0)	137.0 (5.2)	<0.001
WBC (10^9^/L)	6.5 (4.3)	6.5 (4.4)	6.7 (3.7)	0.760
Neutrophil (10^9^/L)	4.4 (3.4)	4.4 (3.6)	4.4 (2.9)	0.323
Lymphocyte (10^9^/L)	1.2 (0.8)	1.1 (0.8)	1.3 (0.8)	0.002
NLR	3.9 (4.1)	4.0 (4.3)	3.3 (3.2)	0.030
Hemoglobin (g/L)	124.0 (29.0)	120.0 (29.0)	133.0 (26.0)	<0.001
PLT (10^9^/L)	92.5 (67.0)	83.0 (61.0)	120.5 (65.0)	<0.001
INR	2.1 (1.0)	2.1 (1.0)	2.0 (0.8)	0.025
**Type of organ failure**				
Circulatory failure	8 (0.9%)	8 (1.2%)	0 (0.0%)	0.118
Renal failure	65 (7.1%)	58 (8.6%)	7 (2.9%)	0.003
Coagulation failure	331 (36.0)	256 (37.9%)	75 (30.7%)	0.045
Liver failure	653 (71.1%)	477 (70.7%)	176 (72.1%)	0.666
Respiratory failure	16 (1.7%)	11 (1.6%)	5 (2.0%)	0.668
Central nervous system failure	41 (4.5%)	32 (4.7%)	9 (3.7%)	0.495
**Severity scores**				
COSSH-ACLF IIs	7.1 (1.3)	7.2 (1.2)	6.7 (1.2)	<0.001
COSSH-ACLFs	7.0 (1.8)	7.2 (1.8)	6.3 (1.5)	<0.001
CLIF-C ACLFs	40.0 (9.3)	40.5 (8.6)	37.6 (10.3)	<0.001
MELDs	26.5 (6.7)	27.1 (7.0)	25.4 (5.5)	<0.001
MELD-Nas	28.0 (9.5)	28.9 (10.6)	26.3 (6.3)	<0.001
TPPMs	0.3 (0.5)	0.4 (0.6)	0.2 (0.3)	<0.001
**LT-free mortality**				
28-day	190 (22.5%)	152 (24.4%)	38 (15.9%)	0.022
90-day	312 (37.0%)	250 (41.1%)	62 (26.3%)	0.001

The data are expressed as medians (interquartile range, IQR), mean ± standard deviation (SD), or the number of patients (%). *P*-value of comparisons between patients with cirrhosis and non-cirrhosis (Student’s *t*-test or Mann–Whitney U-test or χ^2^-test). MAP, mean arterial pressure; GIH, gastrointestinal hemorrhage; HE, hepatic encephalopathy; BI, bacterial infection; SBP, spontaneous bacterial peritonitis; Alb, albumin; ALT, alanine aminotransferase; AST, aspartate aminotransferase; AKP, alkaline phosphatase; TB, total bilirubin; GGT, glutamyl transferase; Cr, creatinine; K, serum potassium; Na, serum sodium; WBC, white blood cell count; NLR, neutrophil-to-lymphocyte ratio; PLT, platelet count; INR, international normalized ratio; COSSH-ACLF II score, Chinese Group on the Study of Severe Hepatitis B-ACLF II score; COSSH-ACLFs, Chinese Group on the Study of Severe Hepatitis B-ACLF score; CLIF-C ACLFs, CLIF-Consortium Acute-on-Chronic Liver Failure score; TPPMs, Tongji Prognostic Predictor Model score; MELDs, Model for End-Stage Liver Disease score; MELD-Nas, MELD-Sodium score; LT, liver transplantation.

### Outcome

**FIGURE 2:**
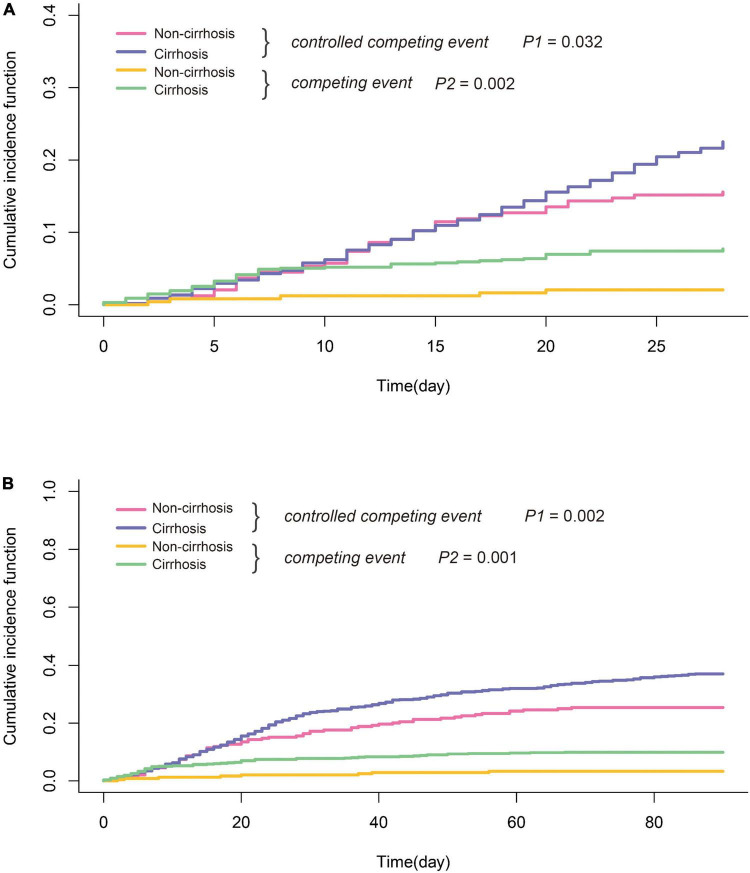
Comparison of short-term survival of HBV-ACLF with or without cirrhosis **(A)** 28-day and **(B)** 90-day; liver transplantation was considered as competing events. Statistical analysis was performed by cumulative incidence function. P1: HBV-ACLF with cirrhosis vs. HBV-ACLF without cirrhosis after controlling for competing risk. P2: HBV-ACLF with cirrhosis vs. HBV-ACLF without cirrhosis without controlling for competing risk.

The median follow-up time of the study was 331 days (data not shown). A total of 312 deaths and 75 LT were recorded during follow-up. As shown in Table 1, 28-day and 90-day mortality in all patients with HBV-ACLF were 22.5 and 37.0%, respectively. Patients with cirrhosis had significantly higher both 28-day and 90-day mortality than those without (24.4/41.1 vs. 15.9/26.3%; 28-day/90-day). Liver transplantation is a common competing event with death in HBV-ACLF. As shown in Figure 2, by a Fine–Gray competing risk model, after controlling the competing event, there was a still significant difference in 28-day and 90-day cumulative survival between the cirrhotic and non-cirrhotic groups (28-day: *p* = 0.032; 90-day: *p* = 0.002). It was also noted that the incidence of LT was more frequent in HBV-ACLF with cirrhosis, concurring with the higher mortality in this subgroup.

### Discrimination analysis

**FIGURE 3:**
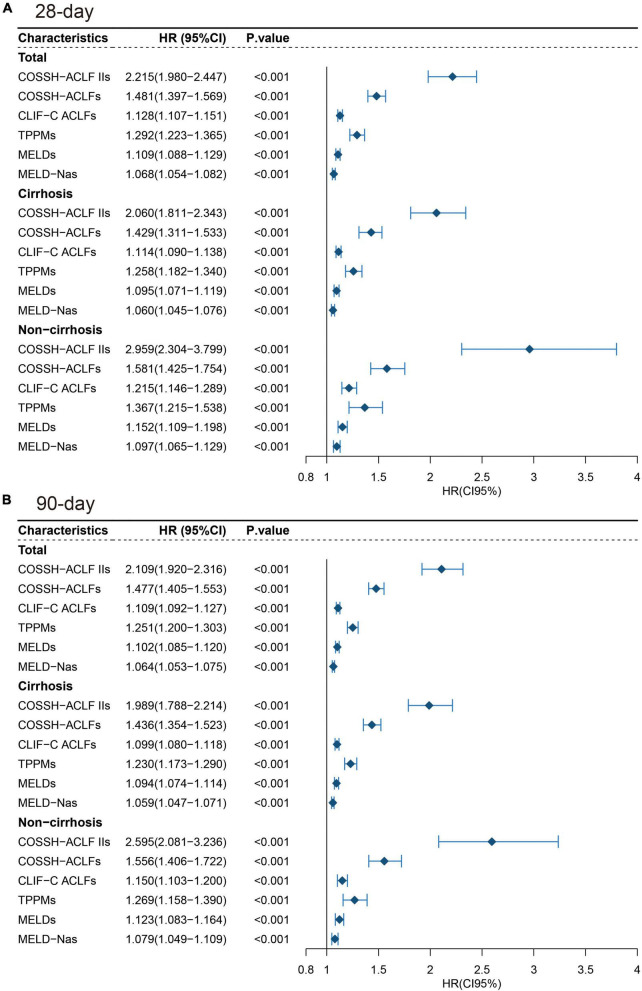
Forest plot for prognostic scores of subgroups of HBV-ACLF. **(A)** 28-day and **(B)** 90-day.

As expected, in a Cox proportional model, each prognostic score was a strong predictor of short-term mortality of HBV-ACLF (Figure 3). The magnitude of risk estimates, as indicated by HR, was greater in patients without cirrhosis for each score, demonstrating a sharper increase in the risk of death with an increment of prognostic scores.

As shown in Table 2, in overall patients, COSSH-ACLF IIs exhibited the highest C-index both in predicting 28-day (0.773) and 90-day outcomes (0.739) in comparison to other scoring models including COSSH-ACLFs, CLIF-C ACLFs, TPPMs, MELDs, and MELD-Nas. COSSH-ACLF IIs remained to have the highest C-index in predicting the prognosis of either patients with cirrhosis or without, except in 90-day outcomes of patients without cirrhosis. By comparison, COSSH-ACLF IIs had better discrimination than other scoring models. The superiority was less significant over COSSH-ACLFs, particularly in the non-cirrhotic group. It was noted that the C-index of each model was shown to be better in patients without cirrhosis than those with. The conclusions remained to be unchanged regardless of whether LT was censored, excluded, or combined with death (Supplementary Tables 1, 2).

**TABLE 2:** Comparisons of C-index of prognostic models in predicting short-term mortality in patients with HBV-ACLF (liver transplantation regarded as censored).

Variates	28-day			90-day		
	C-index	95% CI	*P-*value vs. COSSH-ACLF IIs	C-index	95% CI	*P-*value vs. COSSH-ACLF IIs
**Total HBV-ACLF patients (*n* = 919)**						
COSSH-ACLF IIs	0.773	0.737–0.808	–	0.739	0.710–0.768	–
COSSH-ACLFs	0.751	0.713–0.789	0.044	0.726	0.695–0.756	0.094
CLIF-C ACLFs	0.742	0.704–0.780	<0.001	0.710	0.680–0.740	<0.001
MELDs	0.714	0.673–0.756	<0.001	0.682	0.650–0.715	<0.001
MELD-Nas	0.707	0.667–0.747	<0.001	0.683	0.652–0.714	<0.001
TPPMs	0.721	0.682–0.759	<0.001	0.701	0.669–0.732	<0.001
**All cirrhotic patients (*n* = 675)**						
COSSH-ACLF IIs	0.745	0.703–0.787	–	0.722	0.689–0.755	–
COSSH-ACLFs	0.717	0.671–0.763	0.041	0.706	0.671–0.741	0.094
CLIF-C ACLFs	0.720	0.674–0.765	0.016	0.697	0.662–0.732	0.002
MELDs	0.687	0.638–0.736	<0.001	0.668	0.631–0.705	<0.001
MELD-Nas	0.689	0.642–0.735	0.001	0.674	0.639–0.710	<0.001
TPPMs	0.696	0.651–0.740	0.003	0.686	0.651–0.722	0.003
**All non-cirrhotic patients (*n* = 244)**						
COSSH-ACLF IIs	0.849	0.785–0.913	–	0.767	0.702–0.832	–
COSSH-ACLFs	0.845	0.781–0.909	0.426	0.771	0.708–0.833	0.572
CLIF-C ACLFs	0.800	0.733–0.867	<0.001	0.726	0.662–0.790	0.002
MELDs	0.801	0.730–0.873	0.030	0.717	0.648–0.786	0.023
MELD-Nas	0.772	0.701–0.845	0.001	0.697	0.629–0.765	0.002
TPPMs	0.765	0.681–0.849	0.029	0.699	0.625–0.772	0.056

COSSH-ACLF II score, Chinese Group on the Study of Severe Hepatitis B-ACLF II score; COSSH-ACLFs, Chinese Group on the Study of Severe Hepatitis B-ACLF score; CLIF-C ACLFs, CLIF-Consortium Acute-on-Chronic Liver Failure score; TPPMs, Tongji Prognostic Predictor Model score; MELDs, Model for End-Stage Liver Disease score; MELD-Nas, MELD-Sodium score.

In the study, a total of 919 patients with HBV-ACLF were included under COSSH criteria, of which 434 (47.2%), 550 (59.8%), and 48 (5.2%) patients were ACLF as per EASL-CLIF, APASL, and NASCELD criteria, respectively. As EASL-CLIF and NACSELD criteria only include ACLF with cirrhosis, we compared the predictive performance of CPMs between cirrhotic and non-cirrhotic patients with HBV-ACLF defined by APASL criteria. The baseline characteristics of patients within APASL-ACLF or without are shown in Supplementary Table 3. As shown in Supplementary Table 4, COSSH-ACLF IIs remained to have better discriminative performance than CLIF-C ACLFs, TPPMs, MELDs, and MELD-Nas in predicting both 28-day and 90-day mortality, in either whole population or cirrhosis/non-cirrhosis subgroups. COSSH-ACLF IIs was only better in predicting 28-day outcome in overall patients and patients with cirrhosis when compared to COSSH-ACLFs. Likewise, the C-index of each model in the prediction of 28-day mortality was shown to be better in patients without cirrhosis than those with. However, the trend was not observed in predicting the 90-day outcome.

Then, we compared the NRI and IDI of COSSH-ACLFs, CLIF-C ACLFs, TPPMs, MELDs, and MELD-Nas with COSSH-ACLF IIs, respectively. As shown in Supplementary Table 5, there was no significant difference between CLIF-C ACLFs and COSSH-ACLF IIs. Except for CLIF-C ACLFs, the NRI of COSSH-ACLF IIs significantly improved compared with other models (*p* < 0.05). Moreover, the NRI of patients without cirrhosis is greater than that of cirrhosis, indicating that the prediction efficiency of non-cirrhosis is better. Similarly, as shown in Supplementary Table 6, in the comparison of IDI, there was no significant difference between CLIF-C ACLFs and COSSH-ACLF IIs. Except for CLIF-C ACLFs, COSSH-ACLF IIs had significant improvement in IDI compared with other models (*p* < 0.05). Moreover, the IDI of patients without cirrhosis was greater than that of cirrhosis, also indicating that the prediction efficiency of non-cirrhosis is better.

### Calibration analysis

We further assessed the calibration of scoring models. From an intuitive perspective, COSSH-ACLF IIs and CLIF-C ACLFs represented good calibration, irrespective of 28-day or 90-day outcome and the presence of cirrhosis or not (Supplementary Figure 2). The goodness-of-fit test demonstrated no significant deviation from observed risk in all models except MELDs and TPPMs (Table 3). A quantitative evaluation of model calibration was further by Nagelkerke’s *R*^2^ and the Brier score (Table 4). COSSH-ACLF IIs had the largest Nagelkerke’s *R*^2^ and the least Brier score in predicting 28-day or 90-day outcome in patients with or without cirrhosis, indicating a better calibration. Notably, a larger Nagelkerke’s *R*^2^ and a less Brier score of each model were seen in predicting prognosis in patients without cirrhosis than those with.

**TABLE 3:** Calibration analysis for each score at 28-day and 90-day.

Variates	28-day				90-day			
	H-L test		Calibration plot		H-L test		Calibration plot	
	χ^2^	*p*	Intercept	Slope	χ^2^	*p*	Intercept	Slope
**Total HBV-ACLF patients (*n* = 919)**								
COSSH-ACLF IIs	10.266	0.247	–0.008	1.034	8.403	0.395	–0.020	1.053
COSSH-ACLFs	18.841	0.016	–0.018	1.082	21.148	0.007	–0.025	1.068
CLIF-C ACLFs	3.332	0.912	–0.009	1.040	4.577	0.802	–0.013	1.036
MELDs	17.386	0.026	–0.030	1.135	32.330	<0.001	–0.043	1.118
MELD-Nas	18.717	0.016	–0.029	1.134	18.132	0.020	–0.026	1.071
TPPMs	23.880	0.002	0.050	0.804	14.567	0.068	0.049	0.882
**All cirrhotic patients (*n* = 675)**								
COSSH-ACLF IIs	10.448	0.235	–0.008	1.034	6.999	0.537	–0.014	1.034
COSSH-ACLFs	12.521	0.129	–0.026	1.108	15.544	0.049	–0.030	1.074
CLIF-C ACLFs	7.967	0.437	–0.007	1.027	3.474	0.901	–0.016	1.037
MELDs	19.416	0.013	–0.035	1.143	15.484	0.001	–0.046	1.111
MELD-Nas	10.935	0.205	–0.021	1.087	14.827	0.063	–0.029	1.072
TPPMs	30.528	<0.001	0.116	0.609	13.580	0.093	0.076	0.837
**All non-cirrhotic patients (*n* = 244)**								
COSSH-ACLF IIs	10.954	0.204	–0.010	1.064	7.418	0.492	–0.022	1.084
COSSH-ACLFs	9.615	0.293	–0.012	1.082	16.716	0.033	–0.020	1.075
CLIF-C ACLFs	11.343	0.183	–0.001	1.006	9.419	0.308	–0.006	1.018
MELDs	7.269	0.508	–0.013	1.079	12.435	0.133	–0.036	1.149
MELD-Nas	12.191	0.143	0.011	0.919	4.585	0.801	–0.029	1.113
TPPMs	6.076	0.639	0.014	0.919	10.372	0.240	0.068	0.755

COSSH-ACLF II score, Chinese Group on the Study of Severe Hepatitis B-ACLF II score; COSSH-ACLFs, Chinese Group on the Study of Severe Hepatitis B-ACLF score; CLIF-C ACLFs, CLIF-Consortium Acute-on-Chronic Liver Failure score; TPPMs, Tongji Prognostic Predictor Model score; MELDs, Model for End-Stage Liver Disease score; MELD-Nas, MELD-Sodium score.

**TABLE 4:** Nagelkerke’s *R*^2^ and Brier score of scoring models in patients with HBV–ACLF.

Variates	28-day		90-day	
	Nagelkerke’s *R*^2^ (%)	Brier score	Nagelkerke’s *R*^2^ (%)	Brier score
**Total HBV-ACLF patients (*n* = 919)**				
COSSH-ACLF IIs	27.4	0.133	28.0	0.181
COSSH-ACLFs	22.2	0.140	24.3	0.184
CLIF-C ACLFs	22.6	0.140	20.9	0.194
MELDs	16.8	0.148	15.6	0.201
MELD-Nas	14.3	0.154	16.8	0.200
TPPMs	14.1	0.171	15.9	0.210
**All cirrhotic patients (*n* = 675)**				
COSSH-ACLF IIs	23.0	0.149	25.0	0.194
COSSH-ACLFs	16.8	0.157	21.0	0.198
CLIF-C ACLFs	19.1	0.154	18.4	0.205
MELDs	13.2	0.163	14.3	0.213
MELD-Nas	12.0	0.167	16.3	0.209
TPPMs	10.9	0.192	13.8	0.221
**All non-cirrhotic patients (*n* = 244)**				
COSSH-ACLF IIs	40.8	0.088	30.7	0.143
COSSH-ACLFs	40.4	0.091	27.2	0.146
CLIF-C ACLFs	32.4	0.105	23.1	0.160
MELDs	28.8	0.108	16.3	0.163
MELD-Nas	21.2	0.115	12.8	0.171
TPPMs	18.6	0.110	13.4	0.172

COSSH-ACLF II score, Chinese Group on the Study of Severe Hepatitis B-ACLF II score; COSSH-ACLFs, Chinese Group on the Study of Severe Hepatitis B-ACLF score; CLIF-C ACLFs, CLIF-Consortium Acute-on-Chronic Liver Failure score; TPPMs, Tongji Prognostic Predictor Model score; MELDs, Model for End-Stage Liver Disease score; MELD-Nas, MELD-Sodium score.

### Clinical benefit

**FIGURE 4:**
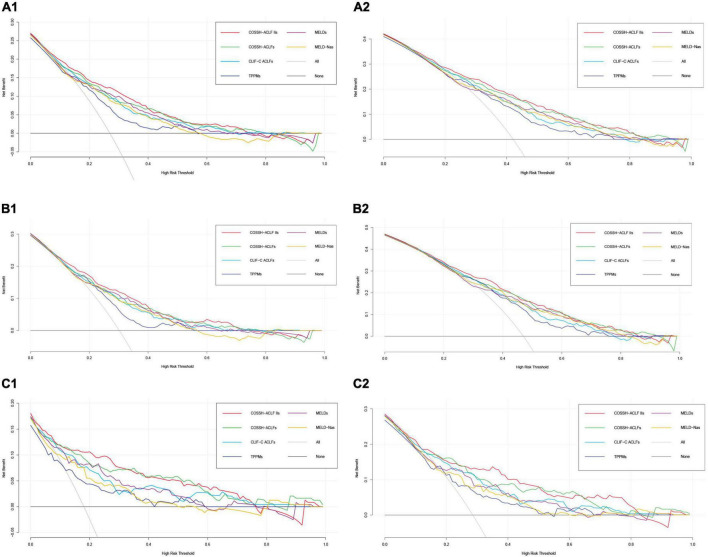
Decision analysis curve in predicting 28/90-day prognosis of overall patients with HBV-ACLF **(A1,A2)**, HBV-ACLF patients with cirrhosis **(B1,B2)**, and HBV-ACLF patients without cirrhosis **(C1,C2)**.

Finally, to evaluate the performance of clinical prognostic models, we used the DCA (Vickers and Elkin, 2006; Vickers, 2008). Of course, this is a theoretical model, which may overestimate or even underestimate the usefulness of the model. As shown in Figure 4, we can find that the six models (COSSH-ACLF IIs, COSSH-ACLFs, CLIF-C ACLFs, TPPMs, MELDs, and MELD-Nas) were all useful between the threshold probability of 30–50%, and the threshold probability range is large in the 90-day prognosis evaluation. In addition, it can be seen from the figure that the clinical net benefits of COSSH-ACLF IIs and COSSH-ACLFs are better than the other three models, especially in patients without cirrhosis.

### Factors associated with short-term prognostication of clinical prediction models in cirrhosis

We first performed a hazard analysis of risk factors associated with poor short-term outcomes in patients with or without cirrhosis, and the findings showed elder age, high INR and neutrophil count, and the presence of HE were independent predictors of 28-day or 90-day LT-free mortality in both HBV-ACLF patients with cirrhosis or without cirrhosis (see Supplementary Table 7). Notably, serum bilirubin was specifically associated with short-term mortality in patients with cirrhosis [28-day and 90-day outcome: HR = 1.002 (1.001–1.002), *p* < 0.001] and blood urea nitrogen in patients without cirrhosis [28-day outcome: HR = 1.111 (1.026–1.204), *p* = 0.010; 90-day outcome: HR = 1.086 (1.017–1.158), *p* = 0.013].

We further performed a subgroup analysis in patients with cirrhosis based on combined complications, such as ascites, hepatic encephalopathy, infections, and gastrointestinal hemorrhage. As shown in Supplementary Tables 8, 9, patients with ascites or infections had a lower discriminative performance of CPMs in predicting 28-day or 90-day LT-free mortality than those without, while the presence of hepatic encephalopathy and gastrointestinal hemorrhage had little impact.

## Discussion

A prior study that developed COSSH-ACLF IIs has shown a high discriminative accuracy of this model in predicting 28-day and 90-day outcomes of overall patients with HBV-ACLF, each C-index exceeding 0.80 (Wu et al., 2018). However, 0.773 (28-day) and 0.739 (90-day) C-indexes were significantly lower in our study. The discrepancy can be attributed to the difference in the constitution of patients with cirrhosis and non-cirrhosis between the two studies. Nevertheless, a relative discriminative superiority of COSSH-ACLF IIs over other scoring systems has been confirmed in both subgroups. In particular, COSSH-ACLF IIs achieved optimal accuracy in predicting 28-day outcomes of patients without cirrhosis, with a C-index close to 0.85. It should be noted that blood urea nitrogen is adopted instead of creatinine in the formula of COSSH-ACLF IIs. Although blood urea nitrogen is a less specific surrogate marker for kidney function than creatinine, creatinine is affected by increased tubular secretion, sarcopenia, and hyperbilirubinemia, and thereby commonly leads to underestimates of kidney function decline in patients with cirrhosis (Velez et al., 2020). In addition to acute kidney injury, other circumstances associated with the elevated level of blood urea nitrogen include gastrointestinal hemorrhage and high levels of catabolism (Zaccherini et al., 2021), which had a negative influence on the outcome of ACLF.

Second, our data clearly demonstrated that each prognostic model had better performance in HBV-ACLF patients without cirrhosis than those with cirrhosis in the study which is consistent with the study of Dong et al. (2020). The limited performance of prognostic scores in ACLF patients with cirrhosis reflects the intrinsic heterogeneity of this subtype, which results from both the wide spectrum of cirrhosis and the diversity of acute precipitating events (Shi et al., 2015a). There is a wide continuum of disease stages during the natural history of cirrhosis. According to the absence or presence of esophageal varices and/or ascites, four clinical stages or statuses of cirrhosis can be identified, each with distinct clinical features and a markedly different prognosis. To be more complicated, progression may be accelerated by the development of other complications such as (re)bleeding, renal impairment (refractory ascites and hepatorenal syndrome), hepatopulmonary syndrome, and sepsis (spontaneous bacterial peritonitis) (Wu et al., 2018; Tang et al., 2021). Thereby, ACLF patients with cirrhosis have varying stages of cirrhosis, which may impact the prognosis. To support it, our prior study has demonstrated that ACLF in patients with cirrhosis with the previous decompensation had higher mortality after 28 days. Our data suggested that the accuracy of CPMs declined with the presence of ascites or infections in patients with cirrhosis. In contrast, the clinical manifestations of ACLF patients with cirrhosis were complicated by the differential effect of hepatic (hepatitis B flare, superimposed infection of HAV or HEV, etc.) and extrahepatic (bacterial infection, upper gastrointestinal bleeding, etc.) precipitating events (Shi et al., 2015a). As shown by our prior study, hepatic-ACLF was classically characterized by liver and coagulation failures, whereas patients with extrahepatic-ACLF displayed diverse phenotypes of organ failures. In addition, 29–44% of ACLF in cirrhosis that occur without an evident precipitating event represent another unique population. Taken together, it is likely that the interaction between precipitating events and underlying cirrhosis leads to complex clinical phenotypes in ACLF patients with cirrhosis. In contrast, ACLF develops in compensatory non-cirrhotic chronic liver diseases that are precipitated by hepatic insults representing a more homogeneous population.

Third, there may be a lack of some key parameters in these available CPMs that reflects the pathophysiology mechanisms of ACLF in cirrhosis. For example, portal hypertension is the initial and main consequence of cirrhosis and is responsible for the majority of its complications. In the compensated phase, portal pressure may be normal or below the threshold level of clinically significant portal hypertension defined by a hepatic venous pressure gradient (HVPG) of at least 10 mmHg. During the transition to decompensated stage, an increase in portal pressure results in the development of complications such as ascites, portal hypertensive gastrointestinal (GI) bleeding, and encephalopathy. Portal hypertension was also observed in ACLF, as shown by increased intrahepatic resistance and HVPG, and associated with mortality (Mehta et al., 2015). Furthermore, relapse of acute variceal bleeding is higher in patients with ACLF and increased with ACLF grades, and in contrast, pre-emptive TIPS placement improved survival (Trebicka et al., 2020; Kumar et al., 2021). Collectively, these findings implied portal hypertension as an essential element in the pathophysiological alterations of ACLF in cirrhosis; however, there is no surrogate marker for portal hypertension in these available CPMs. Second, it has been recognized that systemic inflammation is a major driver of ACLF progression, and WBC or PMN count is usually adopted as the surrogate marker. However, a limitation of WBC or PMN count is that patients with advanced cirrhosis frequently manifest a reduced baseline WBC or PMN count due to hypersplenism, and thereby the systemic inflammation in these patients can be underestimated (Cazzaniga et al., 2009).

In addition, the frequent complications occurring during the natural history of ACLF in cirrhosis render it to be highly dynamic. For example, a prospective–retrospective cohort study enrolling 985 patients from the APASL-ACLF Research Consortium (AARC) database and the Chinese Study Group showed that the cirrhotic group exhibited higher rates of complication, including ascites, infection, and upper gastrointestinal bleeding than the non-cirrhotic group (Chen et al., 2019). The development of complications was demonstrated to be a major risk factor for mortality in HBV-ACLF patients with cirrhosis, with an increase of 1.9–4.7-fold, and 2.6–8.3-fold of death risk in presence of two and three complications, respectively. In line with it, several reports have shown that a high incidence of bacterial or fungal infections occurred secondary to ACLF in patients with cirrhosis and was associated with poor clinical course and high mortality (Jalan et al., 2014a; Fernández et al., 2018; Wong et al., 2021). Also, it has been shown that variceal bleeding was less likely to be controlled by initial endoscopy in patients with ACLF than in patients with non-ACLF cirrhosis, thereby leading to high rebleeding (Trebicka et al., 2020; Kumar et al., 2021).

## Conclusion

In conclusion, our data revealed a differential performance of common scoring models in predicting short-term mortality between HBV-ACLF patients with and without cirrhosis. Common CPMs reached optimal performance in patients without cirrhosis. Therefore, the present study identified a subgroup of HBV-ACLF in which CPMs can be translated into clinical practice, for instance, liver donor allocation and tailoring indications for liver transplantation. However, it remains to be a challenge to predict the short-term prognosis of HBV-ACLF patients with cirrhosis at a precise level, especially those with ascites or infections. More effort should be put on to refine the current prognostication systems for HBV-ACLF with cirrhosis in the future.

## Data availability statement

The raw data supporting the conclusions of this article will be made available by the authors, without undue reservation.

## Ethics statement

The studies involving human participants were reviewed and approved by the Renji Hospital Ethics Committee of Shanghai Jiao Tong University School of Medicine (NCT02457637 and NCT03641872). The patients/participants provided their written informed consent to participate in this study.

## Author contributions

XY, SY, WG, WT, YZo, YHo, QZ, SX, JLiu, RC, LL, BL, XJ, SL, YC, CJ, JZ, LJ, XM, JLi, TL, RZ, XZho, and HR collected and analyzed the data. JSe, YS, HL, GD, XX, XW, XZhe, YHu, JC, ZM, YG, ZQ, FL, XL, JSa, HY, YZe, and WZ designed the research study. XY wrote the manuscript. YS and JSe critically reviewed the manuscript. All authors approved the final version of the manuscript.

## Footnotes

1
http://www.r-project.org/
