# Supplementary material for: Prognosis prediction performs better in patients with non-cirrhosis hepatitis B virus-related acute-on-chronic liver failure than those with cirrhosis
Source: Front Microbiol. 2022 Dec 9;13:1013439. doi: 10.3389/fmicb.2022.1013439 (PMC9780594; doi:10.3389/fmicb.2022.1013439)
Supplement: Supplementary file 1 [file Data_Sheet_1.docx]

**Supplymentary figure 1 The flow chart of study selection under APASL criteria.**

**
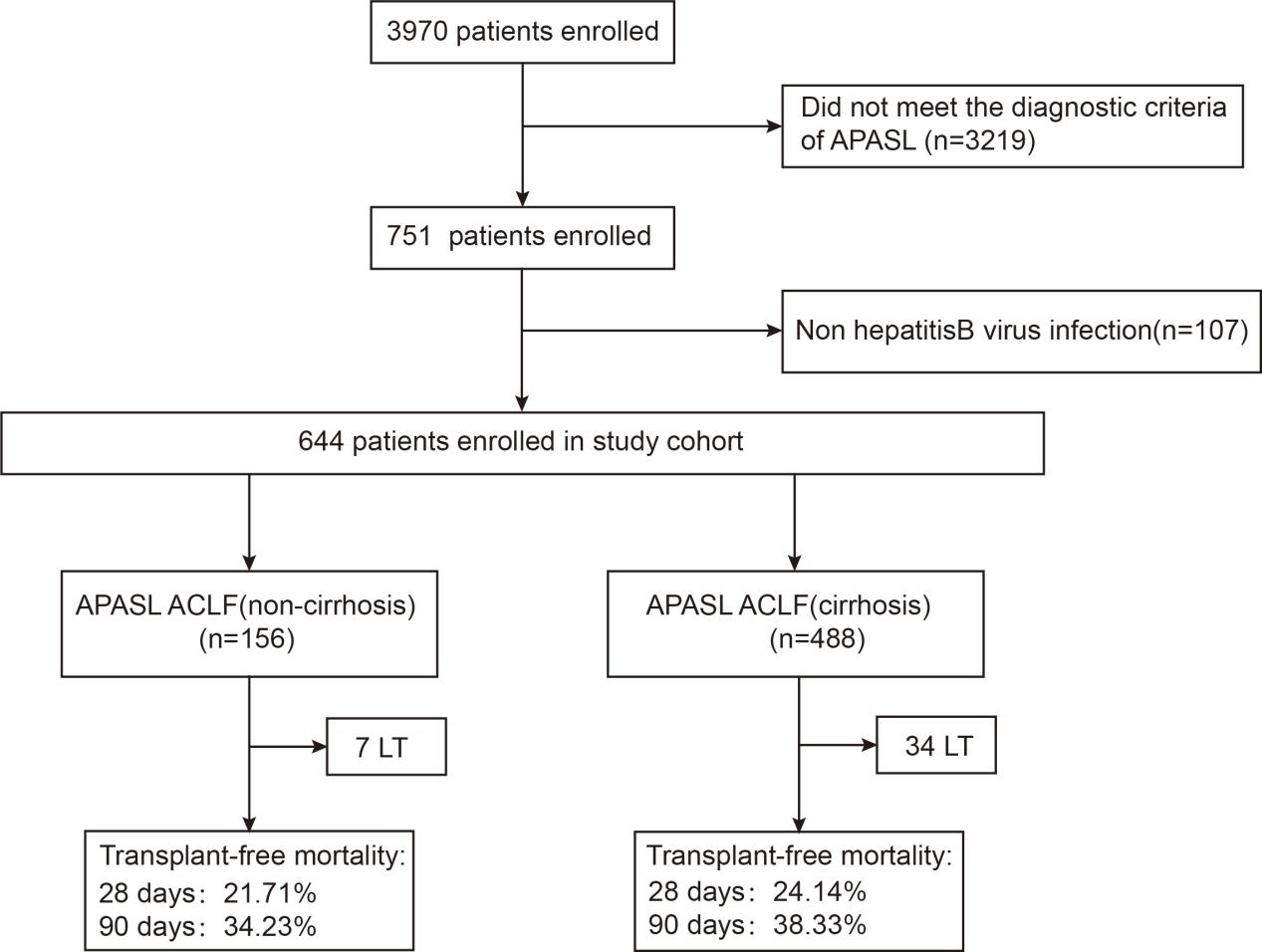
**

LT, Liver transplantation.

**Supplymentary figure 2 Calibration curve for all CPMs(COSSH-ACLF IIs, COSSH-ACLFs, CLIF-C ACLFs, TPPMs, MELDs, MELD-Nas) in predicting 28-day prognosis of overall HBV-ACLF patients, HBV-ACLF patients with cirrhosis and HBV-ACLF patients without cirrhosis.**

**
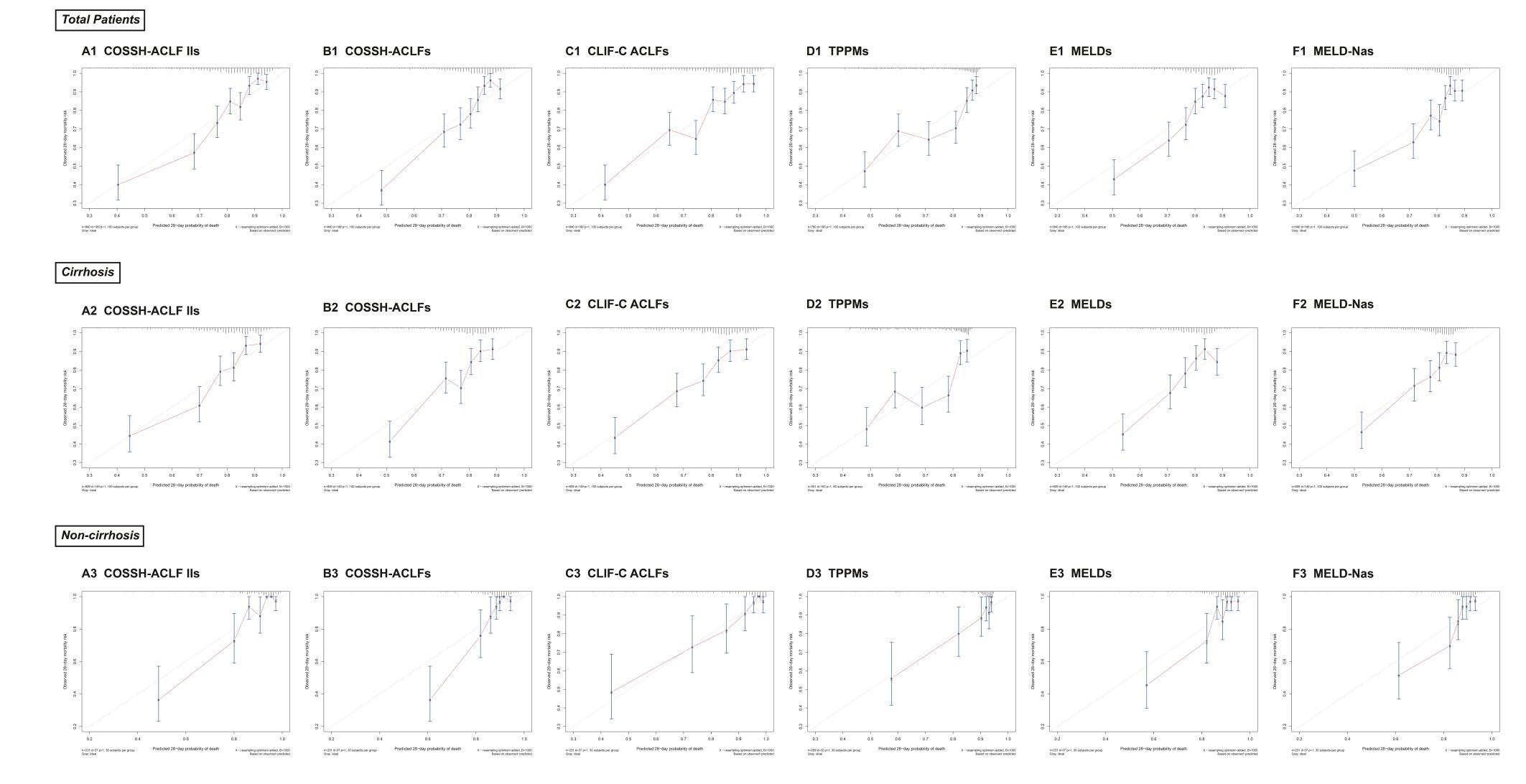
**

**Supplymentary figure 3 Calibration curve for all CPMs(COSSH-ACLF IIs, COSSH-ACLFs, CLIF-C ACLFs, TPPMs, MELDs, MELD-Nas) in predicting 90-day prognosis of overall HBV-ACLF patients, HBV-ACLF patients with cirrhosis and HBV-ACLF patients without cirrhosis.**

**
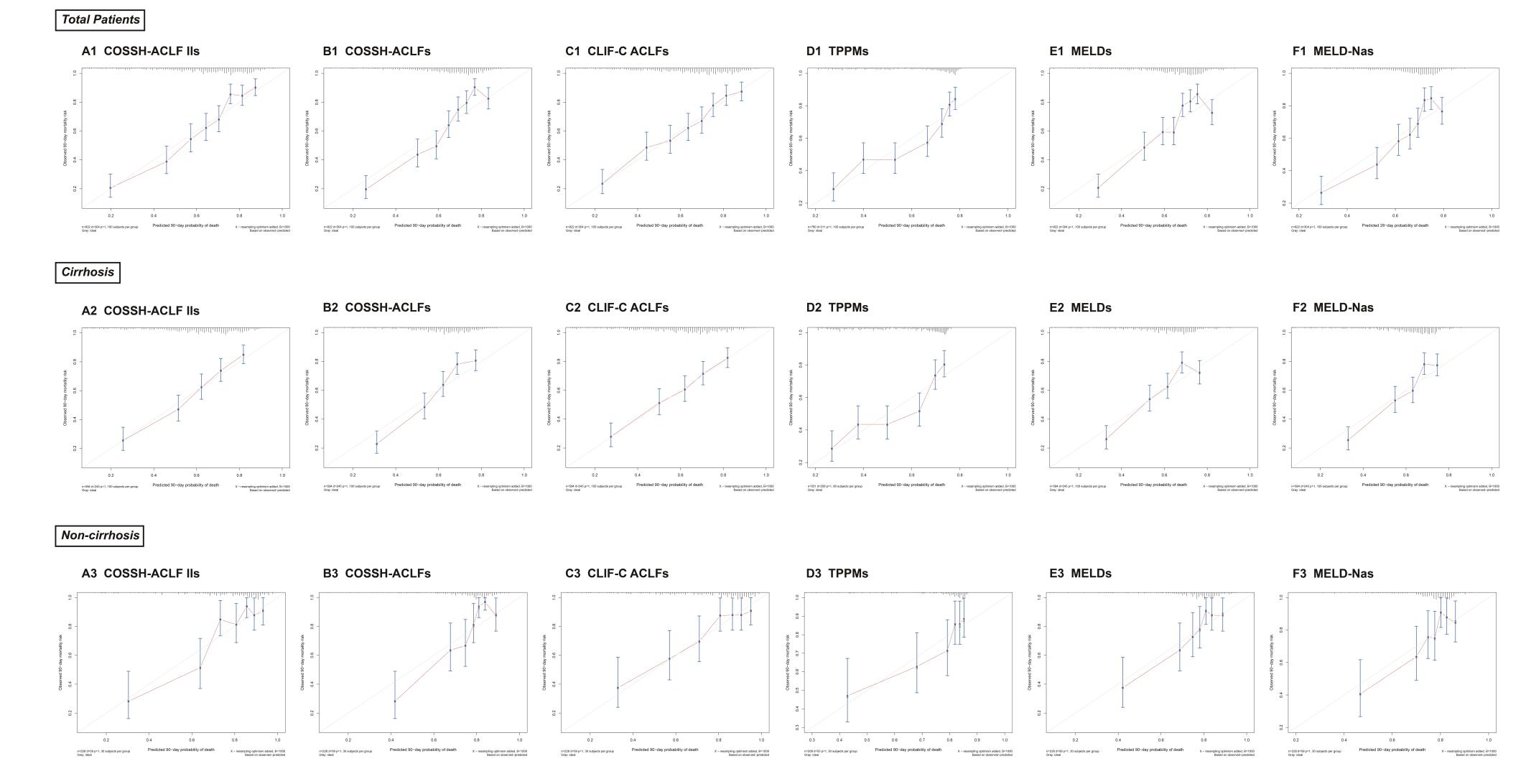
**

**Supplymentary table 1. Comparisons of C-index of prognostic models in predicting short-term mortality in patients with HBV–ACLF under COSSH criteria**

**(Combined liver transplantion and death)**

| **Variates** | **28-day** | | | **90-day** | | |
| --- | --- | --- | --- | --- | --- | --- |
|  | **C-index** | **95% CI** | ***p* value vs. COSSH-ACLF IIs** | **C-index** | **95% CI** | ***p* value vs. COSSH-ACLF IIs** |
| **Total HBV-ACLF patients(n=919)** | | | | | | |
| COSSH-ACLF IIs | 0.775 | 0.739-0.810 | - | 0.740 | 0.711-0.768 | - |
| COSSH-ACLFs | 0.752 | 0.714-0.790 | 0.038 | 0.726 | 0.696-0.756 | 0.090 |
| CLIF-C ACLFs | 0.742 | 0.705-0.810 | <0.001 | 0.710 | 0.680-0.740 | <0.001 |
| MELDs | 0.716 | 0.674-0.758 | <0.001 | 0.683 | 0.650-0.715 | <0.001 |
| MELD-Nas | 0.710 | 0.670-0.750 | <0.001 | 0.685 | 0.653-0.716 | <0.001 |
| TPPM | 0.692 | 0.656-0.727 | <0.001 | 0.679 | 0.650-0.707 | <0.001 |
| **All cirrhotic patients(n=675)** | | | | | | |
| COSSH-ACLF IIs | 0.745 | 0.703-0.787 | - | 0.721 | 0.689-0.754 | - |
| COSSH-ACLFs | 0.717 | 0.671-0.763 | 0.036 | 0.705 | 0.670-0.740 | 0.09 |
| CLIF-C ACLFs | 0.720 | 0.674-0.764 | 0.010 | 0.696 | 0.661-0.730 | 0.001 |
| MELDs | 0.688 | 0.639-0.737 | <0.001 | 0.668 | 0.631-0.705 | <0.001 |
| MELD-Nas | 0.690 | 0.643-0.736 | 0.001 | 0.675 | 0.639-0.710 | <0.001 |
| TPPM | 0.661 | 0.621-0.702 | <0.001 | 0.658 | 0.625-0.691 | <0.001 |
| **All non-cirrhotic patients(n=244)** | | | | | | |
| COSSH-ACLF IIs | 0.853 | 0.789-0.917 | - | 0.770 | 0.706-0.835 | - |
| COSSH-ACLFs | 0.847 | 0.784-0.911 | 0.392 | 0.773 | 0.710-0.835 | 0.548 |
| CLIF-C ACLFs | 0.803 | 0.736-0.870 | <0.001 | 0.728 | 0.663-0.792 | 0.001 |
| MELDs | 0.802 | 0.731-0.873 | 0.023 | 0.718 | 0.649-0.787 | 0.019 |
| MELD-Nas | 0.776 | 0.704-0.848 | 0.001 | 0.699 | 0.631-0.767 | 0.002 |
| TPPM | 0.744 | 0.664-0.824 | 0.019 | 0.692 | 0.625-0.760 | 0.020 |

COSSH-ACLF II score, Chinese Group on the Study of Severe Hepatitis B-ACLF II score; COSSH-ACLFs, Chinese Group on the Study of Severe Hepatitis B-ACLF score; CLIF-C ACLFs, CLIF-Consortium Acute-on-Chronic Liver Failure score; TPPMs, Tongji prognostic predictor model score; MELDs, Model for End-Stage Liver Disease score; MELD-Nas, MELD-sodium score.

**Supplymentary table 2. Comparisons of C-index of prognostic models in predicting short-term mortality in patients with HBV–ACLF under COSSH criteria**

**(Liver transplantation was excluded)**

| **Variates** | **28-day** | | | **90-day** | | |
| --- | --- | --- | --- | --- | --- | --- |
|  | **C-index** | **95% CI** | ***p* value vs. COSSH-ACLF IIs** | **C-index** | **95% CI** | ***p* value vs. COSSH-ACLF IIs** |
| **Total HBV-ACLF patients(n=919)** | | | | | | |
| COSSH-ACLF IIs | 0.774 | 0.738-0.809 | - | 0.740 | 0.711-0.768 | - |
| COSSH-ACLFs | 0.751 | 0.713-0.789 | 0.037 | 0.726 | 0.696-0.756 | 0.088 |
| CLIF-C ACLFs | 0.742 | 0.704-0.780 | <0.001 | 0.710 | 0.680-0.740 | <0.001 |
| MELDs | 0.715 | 0.673-0.756 | <0.001 | 0.683 | 0.650-0.715 | <0.001 |
| MELD-Nas | 0.708 | 0.668-0.748 | <0.001 | 0.685 | 0.653-0.716 | <0.001 |
| TPPM | 0.721 | 0.682-0.759 | <0.001 | 0.700 | 0.669-0.731 | <0.001 |
| **All cirrhotic patients(n=675)** | | | | | | |
| COSSH-ACLF IIs | 0.722 | 0.689-0.754 | - | 0.721 | 0.689-0.754 | - |
| COSSH-ACLFs | 0.705 | 0.670-0.740 | 0.085 | 0.705 | 0.670-0.740 | 0.091 |
| CLIF-C ACLFs | 0.696 | 0.661-0.731 | 0.002 | 0.696 | 0.661-0.730 | 0.001 |
| MELDs | 0.668 | 0.631-0.705 | <0.001 | 0.668 | 0.631-0.705 | <0.001 |
| MELD-Nas | 0.675 | 0.639-0.710 | <0.001 | 0.675 | 0.639-0.710 | <0.001 |
| TPPM | 0.694 | 0.649-0.739 | 0.002 | 0.685 | 0.650-0.720 | 0.003 |
| **All non-cirrhotic patients(n=244)** | | | | | | |
| COSSH-ACLF IIs | 0.768 | 0.703-0.833 | - | 0.770 | 0.706-0.835 | - |
| COSSH-ACLFs | 0.771 | 0.708-0.833 | 0.554 | 0.773 | 0.710-0.835 | 0.548 |
| CLIF-C ACLFs | 0.728 | 0.662-0.791 | 0.002 | 0.728 | 0.663-0.792 | 0.001 |
| MELDs | 0.716 | 0.647-0.785 | 0.020 | 0.718 | 0.649-0.788 | 0.019 |
| MELD-Nas | 0.696 | 0.628-0.763 | 0.002 | 0.699 | 0.631-0.767 | 0.002 |
| TPPM | 0.766 | 0.683-0.850 | 0.025 | 0.700 | 0.627-0.773 | 0.052 |

COSSH-ACLF II score, Chinese Group on the Study of Severe Hepatitis B-ACLF II score; COSSH-ACLFs, Chinese Group on the Study of Severe Hepatitis B-ACLF score; CLIF-C ACLFs, CLIF-Consortium Acute-on-Chronic Liver Failure score; TPPMs, Tongji prognostic predictor model score; MELDs, Model for End-Stage Liver Disease score; MELD-Nas, MELD-sodium score.

**Supplymentary table 3 patient characteristics under APASL criteria**

| **Variables** | **Total HBV-ACLF (n=644)** | **HBV-ACLF with**  **Cirrhosis(n=488)** | **HBV-ACLF without**  **cirrhosis(n=156)** | ***p* value** |
| --- | --- | --- | --- | --- |
| **Age (years)** | 47±11 | 48±11 | 43±12 | <0.001 |
| **Sex** | | | | |
| Male | 550(85.4%) | 421(86.3%) | 129(82.7%) |  |
| Female | 94(14.6%) | 67(13.7%) | 27(17.3%) | 0.271 |
| **MAP(mmHg)** | 89±10 | 89±10 | 88±9 | 0.185 |
| **Etiology** | | | | |
| HBV | 483(75.0%) | 369(75.6%) | 114(73.1%) |  |
| HBV+Alcohol | 84(13.0%) | 67(13.7%) | 17(10.9%) |  |
| HBV+Others | 77(12.0%) | 52(10.7%) | 25(16.0%) | 0.159 |
| **Precipitating event** | | | | |
| Hepatic insults alone | 132(20.5%) | 92(18.9%) | 40(25.6%) |  |
| Mixed with extrahepatic insults | 99(15.4%) | 78(16.0%) | 21(13.5%) |  |
| Extrahepatic insults alone | 191(29.7%) | 155(31.8%) | 36(23.1%) |  |
| Unknown | 222(34.5%) | 163(33.4%) | 59(37.8%) | 0.078 |
| **Complications** | | | | |
| GIH | 24(3.7%) | 24(4.9%) | 0(0.0%) | 0.002 |
| Ascites | 507(78.7%) | 411(84.2%) | 96(61.5%) | <0.001 |
| HE | 106(16.5%) | 76(15.6%) | 30(19.2%) | 0.284 |
| Infection | 245(38.0%) | 197(40.4%) | 48(30.8%) | 0.032 |
| SBP | 13(2.0%) | 13(2.7%) | 0(0.0%) | 0.045 |
| Pneumonia | 179(27.8%) | 138(28.3%) | 41(26.3%) | 0.628 |
| Other infection | 59(9.2%) | 52(10.7%) | 7(4.5%) | 0.020 |
| **Laboratory data** | | | | |
| Alb (g/L) | 30.6(6.7) | 30.0(6.7) | 32.1(5.7) | <0.001 |
| ALT (U/L) | 276.3(612.5) | 212.0(495.6) | 508.0(1016.7) | <0.001 |
| AST (U/L) | 214.8(440.5) | 196.9(361.3) | 359.0(755.1) | <0.001 |
| AKP(U/L) | 147.0(70.0) | 147.0(70.7) | 149.0(65.0) | 0.824 |
| TB (umol/L) | 334.3(257.0) | 332.1(267.2) | 334.6(228.9) | 0.469 |
| GGT (U/L) | 76.3(71.2) | 75.0(71.9) | 79.O(79.6) | 0.676 |
| Cr (umol/L) | 71.0(34.4) | 72.0(36.8) | 69.7(28.1) | 0.271 |
| BUN (mmol/L) | 4.3(3.0) | 4.6(3.3) | 3.7(1.9) | <0.001 |
| K (mmol/L) | 3.8(0.7) | 3.8(0.7) | 3.9(0.7) | 0.096 |
| Na (mmol/L) | 136.7(6.2) | 136.1(6.3) | 137.0(5.9) | <0.001 |
| WBC (109/L) | 6.5(4.5) | 6.5(4.5) | 6.6(4.4) | 0.830 |
| Neutrophil (109/L) | 4.4(3.7) | 4.4(3.8) | 4.3(3.6) | 0.928 |
| Lymphocyte (109/L) | 1.2(0.8) | 1.2(0.8) | 1.3(0.8) | 0.126 |
| NLR | 3.6(4.2) | 3.8(4.2) | 3.3(4.3) | 0.849 |
| Hemoglobin (g/L) | 124.0(28.0) | 121.0(26.0) | 131.0(26.0) | <0.001 |
| PLT (109/L) | 88.0(59.0) | 81.0(57.0) | 111.0(62.0) | <0.001 |
| INR | 2.2(1.0) | 2.2(1.0) | 2.2(0.9) | 0.605 |
| **Type of organ failure** | | | | |
| Circulatory failure | 6(0.9%) | 6(1.2%) | 0(0.0%) | 0.205 |
| Renal failure | 33(5.1%) | 28(5.7%) | 5(3.2%) | 0.212 |
| Coagulation failure | 245(38.0%) | 186(38.1%) | 59(37.8%) | 0.947 |
| Liver failure | 429(66.6%) | 322(66.0%) | 107(68.6%) | 0.548 |
| Respiratory failure | 10(1.6%) | 6(1.2%) | 4(2.6%) | 0.241 |
| Central nervous system failure | 35(5.4%) | 26(5.3%) | 9(5.8%) | 0.832 |
| **Severity scores** | | | | |
| COSSH-ACLF IIs | 7.1(1.3) | 7.2(1.3) | 7.0(1.3) | 0.002 |
| COSSH-ACLFs | 7.0(1.9) | 7.1(2.0) | 6.6(1.7) | 0.001 |
| CLIF-C ACLFs | 40.0(9.8) | 40.2(9.9) | 39.3(11.5) | 0.029 |
| MELDs | 20.4(6.7) | 20.7(7.1) | 19.9(5.9) | 0.217 |
| MELD-Nas | 21.8(9.5) | 22.1(10.5) | 20.5(6.7) | 0.015 |
| TPPMs | 0.4(0.6) | 0.5(0.6) | 0.2(0.5) | <0.001 |
| **LT-free mortality** | | | | |
| 28-day | 145(23.54%) | 112(21.71%) | 33(24.14%) | 0.640 |
| 90-day | 225(37.31%) | 174(34.23%) | 51(38.33%) | 0.499 |

The data are expressed as medians (interquartile range, IQR), mean ± (standard deviation, SD) or number of patients (%).

P value of comparisons between patients with cirrhosis and non-cirrhosis; (Student’s t-test or Mann-Whitney U test or X^2^ test).

MAP, mean arterial pressure; GIH, gastrointestinal haemorrhage; HE, hepatic encephalopathy; BI, bacterial infection; SBP, spontaneous bacterial peritonitis; Alb, albumin; ALT, alanine aminotransferase; AST, aspartate aminotransferase; AKP, alkaline phosphatase; TB, total bilirubin; GGT, glutamyl transferase; Cr, creatinine; K, serum potassium; Na, serum sodium; WBC, white blood cell count; NLR, neutrophil-to-lymphocyte ratio; PLT, platelet count; INR, international normalized ratio; COSSH-ACLF II score, Chinese Group on the Study of Severe Hepatitis B-ACLF II score; COSSH-ACLFs, Chinese Group on the Study of Severe Hepatitis B-ACLF score; CLIF-C ACLFs, CLIF-Consortium Acute-on-Chronic Liver Failure score; TPPMs, Tongji prognostic predictor model score; MELDs, Model for End-Stage Liver Disease score; MELD-Nas, MELD-sodium score; LT, liver transplantation. APASL, Asian Pacific Association for the Study of the Liver.

**Supplymentary table 4. Comparisons of C-index of prognostic models in predicting short-term mortality in patients with HBV–ACLF under APASL criteria**

**(Liver transplantation regarded as censored)**

| **Variates** | **28-day** | | | **90-day** | | |
| --- | --- | --- | --- | --- | --- | --- |
|  | **C-index** | **95% CI** | ***p* value vs. COSSH-ACLF IIs** | **C-index** | **95% CI** | ***p* value vs. COSSH-ACLF IIs** |
| **Total HBV-ACLF patients(n=644)** | | | | | | |
| COSSH-ACLF IIs | 0.775 | 0.736-0.815 | - | 0.742 | 0.709-0.774 | - |
| COSSH-ACLFs | 0.765 | 0.725-0.806 | 0.224 | 0.744 | 0.711-0.776 | 0.575 |
| CLIF-C ACLFs | 0.758 | 0.718-0.799 | 0.035 | 0.724 | 0.691-0.757 | 0.011 |
| MELDs | 0.730 | 0.685-0.775 | 0.001 | 0.705 | 0.669-0.741 | 0.001 |
| MELD-Nas | 0.714 | 0.669-0.758 | <0.001 | 0.687 | 0.651-0.722 | <0.001 |
| TPPM | 0.711 | 0.665-0.756 | <0.001 | 0.691 | 0.654-0.727 | <0.001 |
| **All cirrhotic patients(n=488)** | | | | | | |
| COSSH-ACLF IIs | 0.760 | 0.714-0.806 | - | 0.739 | 0.702-0.776 | - |
| COSSH-ACLFs | 0.744 | 0.695-0.792 | 0.146 | 0.741 | 0.704-0.779 | 0.580 |
| CLIF-C ACLFs | 0.752 | 0.704-0.799 | 0.223 | 0.725 | 0.687-0.763 | 0.060 |
| MELDs | 0.711 | 0.658-0.765 | 0.004 | 0.704 | 0.662-0.745 | 0.006 |
| MELD-Nas | 0.703 | 0.650-0.756 | 0.002 | 0.689 | 0.648-0.730 | <0.001 |
| TPPM | 0.702 | 0.651-0.754 | <0.001 | 0.694 | 0.653-0.735 | <0.001 |
| **All non-cirrhotic patients(n=156)** | | | | | | |
| COSSH-ACLF IIs | 0.820 | 0.747-0.894 | - | 0.751 | 0.686-0.818 | - |
| COSSH-ACLFs | 0.825 | 0.751-0.898 | 0.572 | 0.755 | 0.690-0.820 | 0.569 |
| CLIF-C ACLFs | 0.774 | 0.697-0.851 | 0.004 | 0.717 | 0.651-0.784 | 0.014 |
| MELDs | 0.787 | 0.708-0.867 | 0.090 | 0.704 | 0.633-0.775 | 0.016 |
| MELD-Nas | 0.759 | 0.681-0.837 | 0.009 | 0.684 | 0.614-0.754 | 0.002 |
| TPPM | 0.726 | 0.630-0.821 | 0.031 | 0.665 | 0.586-0.744 | <0.001 |

COSSH-ACLF II score, Chinese Group on the Study of Severe Hepatitis B-ACLF II score; COSSH-ACLFs, Chinese Group on the Study of Severe Hepatitis B-ACLF score; CLIF-C ACLFs, CLIF-Consortium Acute-on-Chronic Liver Failure score; TPPMs, Tongji prognostic predictor model score; MELDs, Model for End-Stage Liver Disease score; MELD-Nas, MELD-sodium score; APASL, Asian Pacific Association for the Study of the Live.

**Supplymentary table 5. Comparisons of NRI of prognostic models in predicting short-term mortality in patients with HBV–ACLF**

| **Variates** | **28-day** | | | **90-day** | | |
| --- | --- | --- | --- | --- | --- | --- |
|  | **NRI vs. COSSH-ACLF IIs** | **95% CI** | ***p* value** | **NRI vs. COSSH-ACLF IIs** | **95% CI** | ***p* value** |
| **Total HBV-ACLF patients(n=919)** | | | | | | |
| COSSH-ACLFs | 0.227 | 0.079-0.345 | <0.001 | 0.150 | 0.016-0.254 | 0.040 |
| CLIF-C ACLFs | -0.004 | -0.141-0.160 | 0.917 | 0.034 | -0.106-0.224 | 0.585 |
| MELDs | 0.292 | 0.179-0.385 | <0.001 | 0.240 | 0.141-0.324 | <0.001 |
| MELD-Nas | 0.298 | 0.155-0.381 | <0.001 | 0.268 | 0.150-0.340 | <0.001 |
| TPPM | 0.216 | -0.001-0.326 | 0.053 | 0.180 | 0.041-0.290 | 0.013 |
| **All cirrhotic patients(n=675)** | | | | | | |
| COSSH-ACLFs | 0.237 | 0.073-0.367 | <0.001 | 0.141 | 0.000-0.282 | 0.040 |
| CLIF-C ACLFs | 0.001 | -0.150-0.218 | 0.817 | 0.029 | -0.108-0.248 | 0.538 |
| MELDs | 0.279 | 0.143-0.380 | <0.001 | 0.233 | 0.122-0.336 | <0.001 |
| MELD-Nas | 0.236 | 0.056-0.351 | <0.001 | 0.207 | 0.073-0.301 | <0.001 |
| TPPM | 0.221 | 0.042-0.351 | 0.027 | 0.175 | 0.022-0.283 | 0.033 |
| **All non-cirrhotic patients(n=244)** | | | | | | |
| COSSH-ACLFs | 0.298 | 0.064-0.537 | 0.020 | 0.193 | -0.028-0.436 | 0.086 |
| CLIF-C ACLFs | -0.145 | -0.417-0.201 | 0.465 | 0.067 | -0.244-0.309 | 0.718 |
| MELDs | 0.384 | 0.042-0.576 | 0.033 | 0.332 | 0.071-0.484 | 0.013 |
| MELD-Nas | 0.507 | 0.286-0.668 | 0.007 | 0.374 | 0.215-0.548 | 0.007 |
| TPPM | 0.224 | -0.098-0.458 | 0.233 | 0.252 | -0.024-0.482 | 0.060 |

COSSH-ACLF II score, Chinese Group on the Study of Severe Hepatitis B-ACLF II score; COSSH-ACLFs, Chinese Group on the Study of Severe Hepatitis B-ACLF score; CLIF-C ACLFs, CLIF-Consortium Acute-on-Chronic Liver Failure score; TPPMs, Tongji prognostic predictor model score; MELDs, Model for End-Stage Liver Disease score; MELD-Nas, MELD-sodium score; NRI, Net Reclassification Index or Improvement

**Supplementary table 6. Comparisons of IDI of prognostic models in predicting short-term mortality in patients with HBV–ACLF**

| **Variates** | **28-day** | | | **90-day** | | |
| --- | --- | --- | --- | --- | --- | --- |
|  | **IDI vs. COSSH-ACLF IIs** | **95% CI** | ***p* value** | **IDI vs. COSSH-ACLF IIs** | **95% CI** | ***p* value** |
| **Total HBV-ACLF patients(n=919)** | | | | | | |
| COSSH-ACLFs | 0.049 | 0.018-0.082 | <0.001 | 0.040 | 0.004-0.066 | 0.020 |
| CLIF-C ACLFs | 0.016 | -0.029-0.052 | 0.917 | 0.031 | -0.004-0.062 | 0.113 |
| MELDs | 0.081 | 0.049-0.111 | <0.001 | 0.080 | 0.044-0.112 | <0.001 |
| MELD-Nas | 0.099 | 0.041-0.144 | <0.001 | 0.085 | 0.040-0.121 | <0.001 |
| TPPM | 0.070 | 0.017-0.110 | 0.013 | 0.059 | 0.009-0.097 | 0.013 |
| **All cirrhotic patients(n=675)** | | | | | | |
| COSSH-ACLFs | 0.048 | 0.011-0.089 | 0.013 | 0.037 | 0.003-0.073 | 0.040 |
| CLIF-C ACLFs | 0.012 | -0.038-0.056 | 0.638 | 0.025 | -0.017-0.060 | 0.213 |
| MELDs | 0.076 | 0.039-0.115 | <0.001 | 0.071 | 0.039-0.105 | <0.001 |
| MELD-Nas | 0.086 | 0.033-0.134 | <0.001 | 0.069 | 0.028-0.109 | 0.007 |
| TPPM | 0.069 | 0.018-0.110 | 0.020 | 0.055 | 0.007-0.092 | 0.027 |
| **All non-cirrhotic patients(n=244)** | | | | | | |
| COSSH-ACLFs | 0.086 | 0.019-0.167 | 0.013 | 0.065 | -0.001-0.144 | 0.053 |
| CLIF-C ACLFs | 0.015 | -0.417-0.201 | 0.465 | 0.046 | -0.039-0.109 | 0.246 |
| MELDs | 0.105 | 0.006-0.205 | 0.040 | 0.102 | 0.022-0.178 | 0.027 |
| MELD-Nas | 0.149 | 0.045-0.240 | 0.013 | 0.135 | 0.060-0.222 | 0.007 |
| TPPM | 0.084 | -0.008-0.164 | 0.073 | 0.093 | -0.001-0.168 | 0.053 |

COSSH-ACLF II score, Chinese Group on the Study of Severe Hepatitis B-ACLF II score; COSSH-ACLFs, Chinese Group on the Study of Severe Hepatitis B-ACLF score; CLIF-C ACLFs, CLIF-Consortium Acute-on-Chronic Liver Failure score; TPPMs, Tongji prognostic predictor model score; MELDs, Model for End-Stage Liver Disease score; MELD-Nas, MELD-sodium score; IDI, Integrated Discrimination Improvement.

**Supplementary table 7. Risk factors associated with 28-day or 90-day LT-free mortality in patients with or without cirrhosis**

| **Variables** | **28-day** | | **90-day** | |
| --- | --- | --- | --- | --- |
|  | **HR(95%CI)** | ***p* value** | **HR(95%CI)** | ***p* value** |
| **Cirrhosis** | | | | |
| Age | 1.025(1.012-1.039) | <0.001 | 1.024(1.013-1.035) | <0.001 |
| TB | 1.002(1.001-1.002) | <0.001 | 1.002(1.001-1.002) | <0.001 |
| INR | 1.261(1.163-1.367) | <0.001 | 1.228(1.146-1.315) | <0.001 |
| Neutrophil count | 1.051(1.017-1.087) | 0.003 | 1.049(1.021-1.077) | <0.001 |
| HE | 1.491(1.025-2.169) | 0.037 | 1.485(1.093-2.017) | 0.011 |
| **Non-cirrhosis** | | | | |
| Age | 1.056(1.024-1.088) | <0.001 | 1.040(1.017-1.064) | 0.001 |
| INR | 1.367(1.156-1.617) | <0.001 | 1.427(1.236-1.648) | <0.001 |
| BUN | 1.111(1.026-1.204) | 0.010 | 1.086(1.017-1.158) | 0.013 |
| HE | 3.930(1.629-9.482) | 0.002 | 1.829(0.781-4.282) | 0.164 |
| Neutrophil count | 1.116(0.962-1.294) | 0.148 | 2.013(1.253-3.235) | 0.004 |

Statistical analysis was performed using multivariable COX hazard model. The variables entered into the multivariate analysis were age, sex, MAP, HE, GIH, infection, ascites, TB (umol/L), WBC(*10E9/L), INR, Cr(umol/L), HBV-DNA(IU/ml), Neutrophil count(*10E9/L), PaO2/FiO2, BUN(mmol/L), PLT(*10E9/L).

Abbreviations: MAP, mean arterial pressure; GIH, gastrointestinal haemorrhage; HE, hepatic encephalopathy; TB, total bilirubin; Cr, creatinine; WBC, white blood cell count; PLT, platelet count; INR, international normalized ratio; BUN, blood urea nitrogen

**Supplementary Table 8. C-index of prognostic models in predicting 28-day mortality in HBV–ACLF patients with cirrhosis stratified by complications**

|  | **Cirrhosis** | | | |
| --- | --- | --- | --- | --- |
|  | **C-index with complications**  **(95% CI)** | ***p* value vs. COSSH-ACLF IIs** | **C-index without complications**  **(95%CI)** | ***p* value vs. COSSH-ACLF IIs** |
| **Ascites** | | | | |
| COSSH-ACLF IIs | 0.714  (0.666-0.763) | - | 0.805  (0.735-0.875) | - |
| COSSH-ACLFs | 0.680  (0.627-0.732) | 0.029 | 0.797  (0.720-0.873) | 0.375 |
| CLIF-C ACLFs | 0.700  (0.648-0.751) | 0.135 | 0.764  (0.687-0.841) | 0.014 |
| MELDs | 0.653  (0.598-0.709) | <0.001 | 0.779  (0.695-0.864) | 0.212 |
| MELD-Nas | 0.653  (0.599-0.706) | 0.002 | 0.777  (0.698-0.856) | 0.183 |
| TPPMs | 0.637  (0.582-0.695) | 0.002 | 0.853  (0.788-0.919) | 0.650 |
| **HE** | | | | |
| COSSH-ACLF IIs | 0.691  (0.618-0.765) | - | 0.728  (0.680-0.777) | - |
| COSSH-ACLFs | 0.721  (0.645-0.796) | 0.847 | 0.686  (0.634-0.0.738) | 0.016 |
| CLIF-C ACLFs | 0.679  (0.599-0.759) | 0.311 | 0.695  (0.643-0.748) | 0.010 |
| MELDs | 0.698  (0.614-0.782) | 0.584 | 0.669  (0.615-0.723) | 0.001 |
| MELD-Nas | 0.707  (0.624-0.791) | 0.684 | 0.667  (0.616-0.718) | 0.002 |
| TPPMs | 0.630  (0.543-0.717) | 0.059 | 0.678  (0.627-0.729) | 0.009 |
| **Infection** | | | | |
| COSSH-ACLF IIs | 0.721  (0.656-0.786) | - | 0.761  (0.712-0.811) | - |
| COSSH-ACLFs | 0.649  (0.578-0.720) | 0.002 | 0.765  (0.712-0.819) | 0.584 |
| CLIF-C ACLFs | 0.713  (0.644-0.783) | 0.331 | 0.721  (0.666-0.777) | 0.002 |
| MELDs | 0.646  (0.571-0.721) | 0.002 | 0.724  (0.667-0.782) | 0.041 |
| MELD-Nas | 0.651  (0.580-0.721) | 0.006 | 0.717  (0.662-0.773) | 0.025 |
| TPPMs | 0.618  (0.542-0.695) | 0.004 | 0.754  (0.695-0.813) | 0.102 |
| **GIH** | | | | |
| COSSH-ACLF IIs | 0.739  (0.596-0.882) | - | 0.742  (0.701-0.784) | - |
| COSSH-ACLFs | 0.644  (0.455-0.834) | 0.065 | 0.721  (0.677-0.766) | 0.087 |
| CLIF-C ACLFs | 0.714  (0.578-0.851) | 0.253 | 0.719  (0.673-0.764) | 0.020 |
| MELDs | 0.690  (0.525-0.855) | 0.207 | 0.692  (0.644-0.739) | 0.001 |
| MELD-Nas | 0.660  (0.484-0.835) | 0.117 | 0.692  (0.647-0.737) | 0.003 |
| TPPMs | 0.683  (0.503-0.863) | 0.304 | 0.695  (0.649-0.740) | 0.002 |

COSSH-ACLF II score, Chinese Group on the Study of Severe Hepatitis B-ACLF II score; COSSH-ACLFs, Chinese Group on the Study of Severe Hepatitis B-ACLF score; CLIF-C ACLFs, CLIF-Consortium Acute-on-Chronic Liver Failure score; TPPMs, Tongji prognostic predictor model score; MELDs, Model for End-Stage Liver Disease score; MELD-Nas, MELD-sodium score; GIH, gastrointestinal haemorrhage; HE, hepatic encephalopathy.

**Supplementary Table 9. C-index of prognostic models in predicting 90-day mortality in HBV–ACLF patients with cirrhosis stratified by complications**

|  | **Cirrhosis** | | | |
| --- | --- | --- | --- | --- |
|  | **C-index with complications**  **(95% CI)** | ***p* value vs. COSSH-ACLF IIs** | **C-index without complications**  **(95%CI)** | ***p* value vs. COSSH-ACLF IIs** |
| **Ascites** | | | | |
| COSSH-ACLF IIs | 0.700  (0.662-0.715) | - | 0.775  (0.717-0.838) | - |
| COSSH-ACLFs | 0.682  (0.643-0.721) | 0.104 | 0.760  (0.694-0.825) | 0.248 |
| CLIF-C ACLFs | 0.676  (0.636-0.715) | 0.010 | 0.748  (0.687-0.809) | 0.034 |
| MELDs | 0.652  (0.612-0.693) | <0.001 | 0.722  (0.649-0.795) | 0.027 |
| MELD-Nas | 0.655  (0.616-0.695) | 0.003 | 0.725  (0.655-0.794) | 0.031 |
| TPPMs | 0.654  (0.612-0.742) | 0.008 | 0.775  (0.707-0.843) | 0.148 |
| **HE** | | | | |
| COSSH-ACLF IIs | 0.706  (0.644-0.768) | - | 0.706  (0.670-0.743) | - |
| COSSH-ACLFs | 0.723  (0.663-0.790) | 0.807 | 0.682  (0.643-0.720) | 0.046 |
| CLIF-C ACLFs | 0.681  (0.611-0.751) | 0.119 | 0.674  (0.636-0.713) | 0.001 |
| MELDs | 0.686  (0.618-0.753) | 0.213 | 0.657  (0.617-0.697) | <0.001 |
| MELD-Nas | 0.698  (0.631-0.764) | 0.384 | 0.661  (0.623-0.699) | 0.002 |
| TPPMs | 0.615  (0.540-0.690) | 0.006 | 0.673  (0.634-0.713) | 0.019 |
| **Infection** | | | | |
| COSSH-ACLF IIs | 0.715  (0.666-0.763) | - | 0.723  (0.681-0.765) | - |
| COSSH-ACLFs | 0.664  (0.612-0.716) | 0.002 | 0.730  (0.685-0.775) | 0.676 |
| CLIF-C ACLFs | 0.689  (0.637-0.741) | 0.024 | 0.697  (0.653-0.742) | 0.013 |
| MELDs | 0.654  (0.600-0.708) | <0.001 | 0.680  (0.633-0.728) | 0.008 |
| MELD-Nas | 0.654  (0.602-0.704) | 0.001 | 0.686  (0.640-0.732) | 0.024 |
| TPPMs | 0.625  (0.569-0.681) | <0.001 | 0.715  (0.665-0.765) | 0.073 |
| **GIH** | | | | |
| COSSH-ACLF IIs | 0.721  (0.601-0.842) | - | 0.722  (0.690-0.755) | - |
| COSSH-ACLFs | 0.660  (0.518-0.802) | 0.095 | 0.710  (0.675-0.744) | 0.149 |
| CLIF-C ACLFs | 0.713  (0.605-0.820) | 0.386 | 0.695  (0.660-0.730) | 0.001 |
| MELDs | 0.673  (0.542-0.805) | 0.160 | 0.675  (0.639-0.711) | <0.001 |
| MELD-Nas | 0.649  (0.510-0.788) | 0.088 | 0.679  (0.644-0.713) | <0.001 |
| TPPMs | 0.725  (0.592-0.814) | 0.222 | 0.686  (0.650-0.723) | 0.003 |

COSSH-ACLF II score, Chinese Group on the Study of Severe Hepatitis B-ACLF II score; COSSH-ACLFs, Chinese Group on the Study of Severe Hepatitis B-ACLF score; CLIF-C ACLFs, CLIF-Consortium Acute-on-Chronic Liver Failure score; TPPMs, Tongji prognostic predictor model score; MELDs, Model for End-Stage Liver Disease score; MELD-Nas, MELD-sodium score; GIH, gastrointestinal haemorrhage; HE, hepatic encephalopathy
